# Improvement in cardiovascular function after atrial fibrillation ablation in heart failure: association with ECG and echocardiographic variables

**DOI:** 10.1093/europace/euag086

**Published:** 2026-05-16

**Authors:** Nikhil Ahluwalia, Shohreh Honarbakhsh, Vijay Shyam-Sundar, Abhishek Joshi, Hakam Abbass, Anthony Chow, Mehul Dhinoja, Ross J Hunter, Steffen Petersen, Guy Lloyd, Richard J Schilling

**Affiliations:** William Harvey Research Institute, Faculty of Medicine and Dentistry, Queen Mary University of London, Charterhouse Square, London EC1M 6BQ, United Kingdom; William Harvey Research Institute, Faculty of Medicine and Dentistry, Queen Mary University of London, Charterhouse Square, London EC1M 6BQ, United Kingdom; William Harvey Research Institute, Faculty of Medicine and Dentistry, Queen Mary University of London, Charterhouse Square, London EC1M 6BQ, United Kingdom; William Harvey Research Institute, Faculty of Medicine and Dentistry, Queen Mary University of London, Charterhouse Square, London EC1M 6BQ, United Kingdom; William Harvey Research Institute, Faculty of Medicine and Dentistry, Queen Mary University of London, Charterhouse Square, London EC1M 6BQ, United Kingdom; William Harvey Research Institute, Faculty of Medicine and Dentistry, Queen Mary University of London, Charterhouse Square, London EC1M 6BQ, United Kingdom; William Harvey Research Institute, Faculty of Medicine and Dentistry, Queen Mary University of London, Charterhouse Square, London EC1M 6BQ, United Kingdom; William Harvey Research Institute, Faculty of Medicine and Dentistry, Queen Mary University of London, Charterhouse Square, London EC1M 6BQ, United Kingdom; William Harvey Research Institute, Faculty of Medicine and Dentistry, Queen Mary University of London, Charterhouse Square, London EC1M 6BQ, United Kingdom; William Harvey Research Institute, Faculty of Medicine and Dentistry, Queen Mary University of London, Charterhouse Square, London EC1M 6BQ, United Kingdom; William Harvey Research Institute, Faculty of Medicine and Dentistry, Queen Mary University of London, Charterhouse Square, London EC1M 6BQ, United Kingdom

## Introduction

Catheter ablation (CA) of atrial fibrillation (AF) is an effective heart failure (HF) therapy for patients with left ventricular systolic dysfunction (LVSD).^1^ However, not all patients derive benefit from the procedure despite achieving sinus rhythm.^2,3^ Most reported predictors are retrospective, show limited negative predictive value (NPV), or confound AF recurrence with nonresponse.^3^

The Restitution Threshold Index (RTI) is a novel ECG-based measure that quantifies the burden of short R–R intervals during rate-controlled AF at rest, previously associated with LVSD in the setting of persistent AF.^4^ This prospective study aimed to evaluate the predictive utility of RTI and a novel echocardiographic marker, contractile reserve (CR), in identifying patients most likely to demonstrate comprehensive cardiac functional recovery after CA.^5^

## Methods

### Study population

Consecutive patients with persistent AF and LVSD (LVEF <50%) undergoing first-time CA between January 2022 and September 2023 were prospectively screened. Patients with known, pre-existing ischaemic or non-ischaemic cardiomyopathy or cardiac devices *in situ* were eligible.

The study received approval from the UK National Research Ethics Committee (21/SW/0135) and was prospectively registered at clinicaltrials.gov (NCT04987723).

### ECG and echocardiography assessment

Before CA, participants underwent supervised 10-min Holter ECG recordings in AF to derive RTI, defined as the percentage of R–R intervals <660 ms. Contractile reserve was determined from baseline exercise–stress echocardiography as the absolute increase in LVEF from rest to peak stress.

### Cardiac function assessment

Cardiac function was assessed at baseline and 6-month follow-up using

1. LVEF on echocardiography
2. Peak VO_2_ on cardiopulmonary exercise testing
3. NT-proBNP levels

Responders were defined as those with significant improvement in ≥2/3 parameters, based on the Universal Definition of Heart Failure and thresholds established in prior literature (LVEF, ≥10% increase or to ≥50%; VO2max, ≥6% or exceeding predicted, reduction in NTpro-BNP).^2,3,6,7^ The association between RTI and CR with responder status was the primary outcome. Repeat CA restarted the follow-up clock.

### Statistical analysis

Discrimination was quantified using the area under the receiver-operating characteristic curve (AUROC) analysis. Restitution Threshold Index and CR were entered into a Bayesian logistic regression model adjusted for age and sex and reported as mean coefficient [95% Highest Density Interval (HDI)]. A Bonferroni correction (*P* < 0.017) was applied to account for multiple cardiac function endpoints.

## Results

### Study cohort

A total of 53 patients (mean age 59 ± 12 years, 15% female) were enrolled of 98 patients screened. Median duration of persistent AF was 10 (5–14) months, and mean baseline LVEF was 34 ± 9%. After the index CA, patients were followed for a mean of 16 ± 5 months; eight (15.1%) underwent repeat left atrial CA. Two participants did not complete follow-up, and four were not in sinus rhythm at follow-up; they were excluded from endpoint analysis.

### Cohort and outcomes

**Figure 1.**
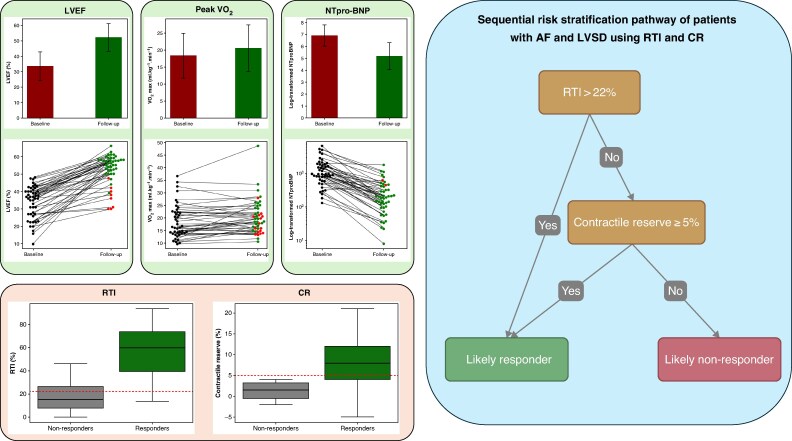
Catheter ablation improves cardiac function in patients with left ventricular dysfunction, although individual response can be variable (green). Novel ECG and echocardiographic parameters are associated with response (red) and their sequential assessment is proposed (blue).

All three cardiac parameters improved significantly in sinus rhythm on a cohort level (*Figure 1*). The absolute change in LVEF correlated with baseline RTI (r = 0.35, *P* = 0.016) and CR (r = 0.39, *P* = 0.008). Forty-three of 47 (91.5%) participants improved their cardiac function in at least two parameters in sinus rhythm and were classified as responders.

### Predictors of response

Responders and nonresponders were comparable in demographics, atrial or ventricular chamber dimensions on echocardiography, and cardiac function parameters at baseline. Six of 10 (66.7%) participants with a known pre-existing HF aetiology before AF onset met responder status.

Responders had higher RTI (58 ± 22% vs. 19 ± 20%, *P* = 0.001) and a greater CR (8 ± 6% vs. 1 ± 3%, *P* = 0.03) than nonresponders. There was no difference in the mean heart rate between responders and nonresponders during AF (86 ± 14bpm vs. 81 ± 9bpm, *P* = 0.50) or in the peak heart rate during exercise testing (142 ± 17bpm vs. 124 ± 24bpm, *P* = 0.06).

### Predictive performance of RTI and CR

An RTI >22% had a positive predictive value (PPV) of 0.98 and a sensitivity of 0.95. The NPV was 0.60. Restitution Threshold Index had an AUROC of 0.91 for predicting response, and when sequentially combined with CR, the AUROC increased to 0.97, with an NPV of 1.0. A positive association was observed between RTI (mean coefficient 0.13, 95% HDI: 0.04 to 0.21), CR (mean coefficient 0.42, 95% HDI: 0.08 to 0.77), and responder status after CA, as determined by logistic regression analysis. There was no significant difference between participants who had an AF recurrence compared to those who did not in terms of baseline RTI (49 ± 31% vs. 54 ± 24%, *P* = 0.562) or CR [10% (3, 14) vs. 6 (3, 12) *P* = 0.390]. A sensitivity analysis showed that participants who were in AF at follow-up did not significantly improve their LVEF (*P* = 0.24), peak VO2 (*P* = 0.39), or NT-proBNP level (*P* = 0.55).

## Discussion

Restitution Threshold Index and CR are simple, inexpensive, and clinically accessible tools that demonstrate excellent discriminatory ability, as indicated by AUROC analysis in this exploratory study, and are associated with responder status in logistic regression. The ECG and echocardiographic variables identified patients likely to exhibit substantial cardiac functional recovery after AF CA and may characterize the AF-mediated cardiomyopathic process—the most common form of arrhythmia-induced cardiomyopathy.^8^ Retrospective studies that retain patients with AF recurrence may confound their nonresponder status by association with predictors of AF recurrence, which are not upheld in rhythm-dependent analyses. We excluded these participants from the primary endpoint analysis in this exploratory study of the AF-mediated cardiomyopathy phenomenon specifically and report the outcome of participants with AF recurrence separately, in whom responder status is unknown. As a result, our nonresponder cohort was small, and findings are vulnerable to overfitting, warranting larger studies and external validation of these hypothesis-generating results.

Ventricular late gadolinium enhancement on MRI, while mechanistically informative, has shown limited NPV and requires specialized infrastructure.^9,10^ The simplicity of RTI and CR allows their use in routine practice, offering a pragmatic framework for identifying patients with potentially reversible AF-mediated myocardial dysfunction.

## Data Availability

Study data will be shared on reasonable request to the corresponding author.
